# Reliability and validity of the Mental Health Self-management Questionnaire among Chinese patients with mood and anxiety disorders

**DOI:** 10.3389/fpsyt.2022.952951

**Published:** 2022-07-29

**Authors:** Mengmeng Wang, Jingjun Wang, Ya Wang, Xia Huang, Yalin Huang, Junqiang Huang, Yan Feng, Xiaolin Li

**Affiliations:** ^1^West China School of Nursing and West China Hospital, Sichuan University, Chengdu, China; ^2^Department of Nursing, West China Hospital and West China School of Nursing, Sichuan University, Chengdu, China; ^3^Mental Health Centre, West China Hospital, Sichuan University, Chengdu, China; ^4^Chengdu Dekang Hospital/Chengdu Psychiatric Hospital, Chengdu, China; ^5^Longhua Hospital Shanghai University of Traditional Chinese Medicine, Shanghai, China

**Keywords:** mental health, self-management, mood disorders, anxiety disorders, reliability, validity

## Abstract

**Background:**

Self-management plays an important role in promoting and restoring mental health for individuals with mental health issues. However, there is no valid and reliable Chinese tool assessing the self-management behaviors of people with mood and anxiety disorders. This study aimed to develop a Chinese version of the Mental Health Self-management Questionnaire (MHSQ-C) and to verify its psychometric properties.

**Methods:**

A total of 440 potential participants were recruited by convenience sampling from June to August 2020. Item analysis and analyses of internal consistency, test-retest reliability, content validity, construct validity and criterion validity were performed.

**Results:**

Data from 326 participants were used. Three factors obtained *via* principal component analysis and varimax rotation explained 53.68% of the total variance. The average content validity index was 0.99. The Cronbach’s α coefficient (total: 0.874, clinical: 0.706, empowerment: 0.818, vitality: 0.830) and test-retest reliability (ICC: total: 0.783, 95% confidence interval (CI) [0.616, 0.882], clinical: 0.525, 95% CI [0.240, 0.725], empowerment: 0.786, 95% CI [0.622, 0.884], vitality: 0.748, 95% CI [0.564, 0.862]) were good. The MHSQ-C was well correlated with the Partners in Health scale and showed no floor or ceiling effect.

**Discussion:**

The MHSQ-C is a reliable and valid tool to evaluate the self-management strategies of patients with mood and anxiety disorders.

## Introduction

Mood disorders (depression and bipolar disorder) and anxiety disorders are common mental health issues worldwide. Many studies have examined the lifetime or 12-month prevalence of mood and anxiety disorders among the populations of various countries. In the United States, 20.29% of the population was reported to have mood and anxiety disorders in the last 12 months (1). In Argentina, there is a lifetime and 12-month prevalence of mood and anxiety disorders of 40.4 and 20.1%, respectively, in public primary healthcare centers (2), and in China, those numbers are 15.0 and 9.1%, respectively, among Chinese community dwellers (3). Mood and anxiety disorders are associated with high costs to the individual and society, including a low level of education attainment and unstable employment (4), decreased health-related quality of life (5, 6), increased mortality risk (7), and increased economic burden (8); these disorders are the leading causes of overall disease burden (9, 10).

Pharmacological and psychological therapies are the main treatment methods for people with mental disorders, but the treatment rates remain low because of various barriers, such as perceived social stigma and the availability of professional help (11, 12). A study estimated the treatment proportion of individuals with 12-month anxiety disorders in 21 countries, showing that 27.6% of these individuals received any treatment, but that only 9.8% received possibly adequate treatment (13). Lower treatment rates were found for lower-income countries: 21.9% of respondents with any 12-month disorder sought treatment within the past 12 months in Japan (14), and 13.7% of respondents with a 12-month mental disorder received 12 months of treatment in Saudi Arabia (15). Moreover, relapses are also possible even with adequate treatment. For example, the estimated cumulative recurrence rate of anxiety was 2.1% at 1 year, 6.6% at 5 years, 10.6% at 10 years, and 16.2% at 20 years (16). For major depressive disorder (MDD), the cumulative recurrence rate was 4.3% at 5 years, 13.4% at 10 years and 27.1% at 20 years (17). To reduce symptoms and relapses, self-management is recommended by mental health guidelines as a complementary strategy to pharmacotherapy and psychotherapy to prompt recovery (18, 19).

Self-management is a life-long task encompassing medical and emotional management to assume personal skills such as problem solving, decision making, and resource utilization (20, 21). A body of studies has pointed out the benefits of self-management for people with mental disorders, including reducing symptom severity (22), improving global functioning (23, 24), increasing quality of life (25, 26), and prompting recovery, hope and self-efficacy (27). To prompt the recovery of individuals with mental disorders, it is important to evaluate and support their self-management behaviors as outcomes of their self-management. However, most research has selected symptoms, quality of life, or social functioning as outcome indicators for self-management interventions.

Moreover, the Illness Management and Recovery Scales (IMRS) (28–30), the Mental Health Recovery Measure (MHRM) (31–33), the Patient Activation Measure for Mental Health (PAM-MH) (34, 35), the Recovery Assessment Scale (RAS) (36–38), the Mental Health Confidence Scale (MHCS) (39–41), the Task-Specific Self-Efficacy Scale (TSSES) (42), and the Schizophrenia Self-Management Instrument Scale (SSMIS) (43) are used widely in self-management programs in the mental health field, although most of them were not designed to assess self-management behaviors. Furthermore, only the TSSES and SSMIS were developed using Chinese patients; the IMRS and RAS had Chinese versions. The characterization of these tools has been mentioned in our previous research (44). A reliable and valid measurement assessing self-management behaviors is essential for assessing self-management among mood and anxiety disorders in China.

The Mental Health Self-management Questionnaire (MHSQ) was developed by Coulombe et al. to measure the use of self-management strategies among patients with mood and anxiety disorders (45). The MHSQ is a self-report scale with 18 item responses ranging from 0 (never used) to 4 (very often used). The MHSQ was reported to have satisfactory reliability, examined by Cronbach’s α (Clinical = 0.69, Empowerment = 0.81, Vitality = 0.75) and the test-retest reliability of each factor using the intraclass correlation coefficient (ICC) (Clinical: ICC = 0.78; Empowerment: ICC = 0.76; Vitality: ICC = 0.85) (45). The validity of the original MHSQ was examined for its content validity, concurrent validity, convergent validity, and discriminant validity from the recovery concept (45). The Japanese version, the MHSQ-J, has also been reported to have good reliability and validity (46).

Previous studies indicate that there are no measures of self-management behaviors for mood and anxiety in China. A reliable and valid measurement is important to guide clinical practice. Therefore, the aim of this study was to develop a Chinese version of the MHSQ (MHSQ-C) and to verify its psychometric properties among people with mood and anxiety disorders.

## Materials and methods

### Study design

A cross-sectional design was used to evaluate the psychometric properties of the MHSQ-C. This study was reported following the STrengthening the Reporting of OBservational Studies in Epidemiology (STROBE) checklist for observational research (47).

### Development of the Chinese version of the Mental Health Self-management Questionnaire

**FIGURE 1:**
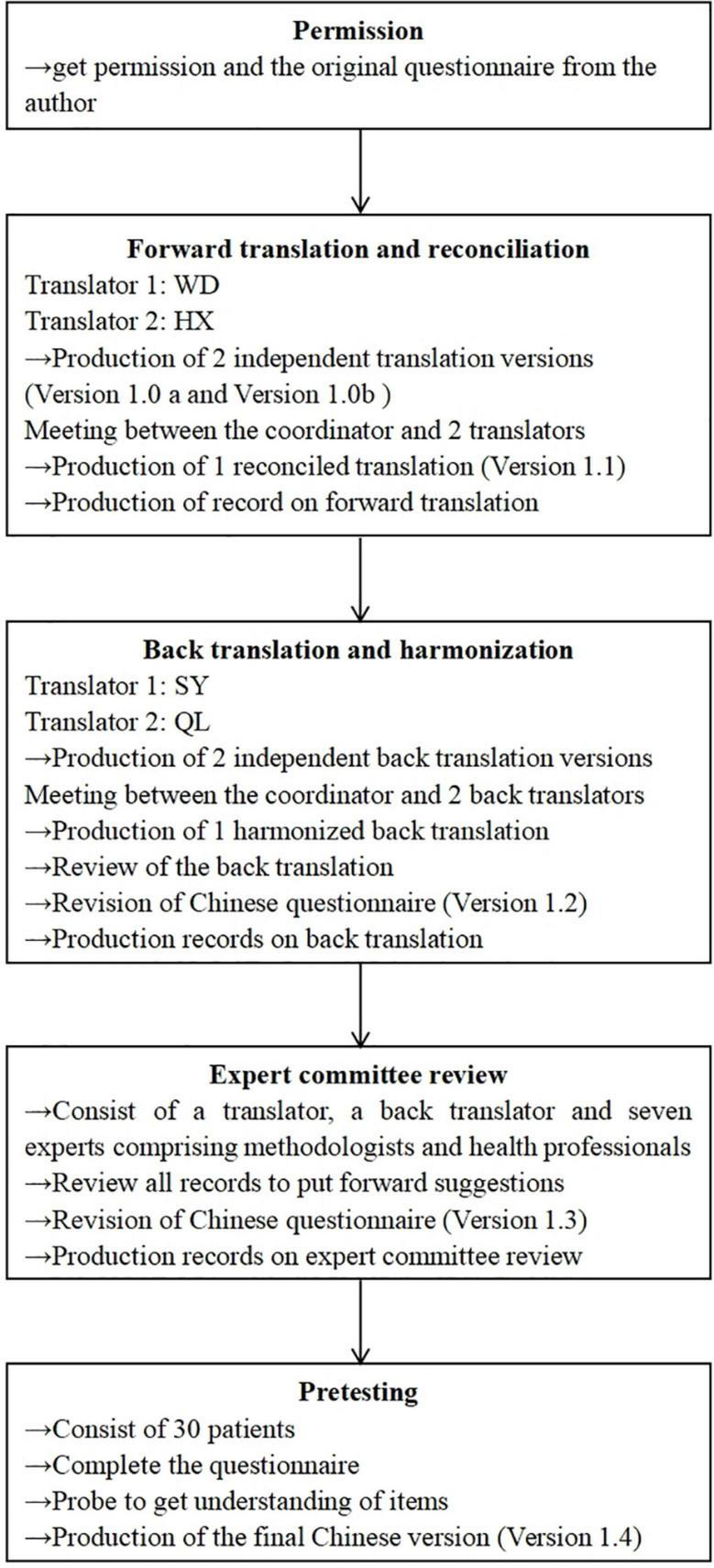
The process of developing the Chinese Mental Health Self-management Questionnaire.

The original English version of the MHSQ consists of 18 items measuring the frequency of self-management strategies (Supplementary Appendix A). The development procedures of the MHSQ-C were conducted in accordance with the guidelines and principles of Wild et al. (48) and Beaton et al. (49) (Figure 1).

#### Permission for the translation and acquisition of the English version of the Mental Health Self-management Questionnaire

The first author obtained permission from the author of the original MHSQ by e-mail for its acquisition and translation into Chinese.

#### Forward translation and reconciliation

Two translators separately and independently translated the English version of the MHSQ into Chinese (Ver 1.0a and Ver 1.0b). Both forward translators are native speakers of Chinese, are proficient in English and have different profiles. Details about the translators and experts can be found in Supplementary Appendix B. Through comprehensive discussion between the coordinator and the translators, Ver 1.0a and Ver 1.0b were reconciled into one forward translation (Ver 1.1).

#### Back translation and harmonization

Two bilingual translators (Supplementary Appendix B) who had not seen the original English version of the MHSQ independently produced two back translations of Ver 1.1 into English. Then, the coordinator compared the two back-translated versions with the original English version. Any discrepancies among the original and back-translated versions were reviewed and discussed by the coordinator and back translators to determine whether revisions to the Chinese wording were necessary. Ver 1.1 was revised in consultation with the forward translators when modifications were necessary until the two back translators agreed with each other, and the second Chinese version (Ver 1.2) was subsequently created.

#### Expert committee review

The committee consisted of one forward translator, one back translator and seven experts comprising methodologists and health professionals (50). The experts were provided with detailed records on the forward and back translation steps and then made recommendations for Ver 1.2. The coordinator recorded the recommendations and reviewed and discussed them with the research team worker, resulting in the creation of the third Chinese version (Ver 1.3).

#### Pretesting

A total of 30 patients were tested by convenience sampling to ensure that the Chinese version retained its equivalence in an applied situation. Each subject completed the questionnaire and was interviewed to probe what he or she thought was meant by each questionnaire item and the chosen response. The researcher recorded the comments in the process, discussed them with members of the research group, and created the fourth Chinese version (Ver 1.4).

### Reliability and validity of the Chinese version of the Mental Health Self-management Questionnaire

The process to evaluate reliability and validity followed the guidelines for translation, adaptation and validation of instruments (51). Item analysis was used to assess the performance of individual items in the MHSQ-C. The methods of item analysis included critical ratio, correlation analysis, Cronbach’s alpha coefficient, communalities and factor loading. The reliability of the MHSQ-C was evaluated based on the internal consistency (Cronbach’s alpha coefficient) and test-retest reliability (ICC) to assess the consistency, stability and reliability of the results. A previous study used response data that were provided within a period of 8–20 days between the test and retest (46). The retest interval is generally 10–14 days (52). Considering the feasibility of the study, participants completed the MHSQ-C twice with an inter-test interval of approximately 1–2 weeks. Internal consistency describes the extent to which all the items in a test measure the same concept or construct (homogeneous). The validity of the MHSQ-C was confirmed based on the content validity, construct validity and criterion validity to assess the extent to which the instrument accurately measured what was intended.

### Participants and sample size

A total of 440 potential subjects were recruited from the mental health outpatient and inpatient departments in the general hospital by convenience sampling from June 2020 to August 2020. The inclusion criteria for the participants were as follows: (a) had anxiety or mood disorder including bipolar affective disorder (F31), depressive episodes (F32), recurrent depressive disorders (F33), phobic anxiety disorders (F40) or other anxiety disorders (F41) as diagnosed by a psychiatrist according to the International Classification of Diseases-10th edition (ICD-10); (b) were diagnosed more than 3 months prior; (c) were no younger than 13 years old; and (d) were able to understand the questionnaires. Subjects were excluded if they (a) were also diagnosed with schizophrenia, eating disorders and alcohol or substance abuse or (b) refused to participate in the study. The sample size was calculated based on a rule of thumb of at least 10 respondents for each item in factor analysis (52). We assumed a 20% nonresponse rate, so the minimum sample size was 216.

### Instruments

#### Participant characteristics

The sociodemographic and clinical information of participants was collected, including age, sex, ethnicity, marital status, income, educational background, somatic comorbidities, mental comorbidities, diagnosis, number of recurrences, severity of depression and anxiety, period from first diagnosis to investigation and number of hospitalizations due to mental health problems. The severities of depression and anxiety were assessed using the Patient Health Questionnaire-9 (PHQ-9) and the General Anxiety Disorder-7 (GAD-7), respectively. The PHQ-9 is a self-report scale with a response to each of nine items ranging from 0 (not at all) to 3 (nearly every day) (53). The GAD-7 is also a self-report scale with each of seven item responses ranging from 0 (not at all) to 3 (nearly every day) (54). The Chinese versions of the PHQ-9 and GAD-7 are brief, well-validated tools with Cronbach’s alpha values of 0.86 and 0.93 among the general Chinese population and Chinese medical students, respectively (55, 56).

#### Partners in health scale

The partners in health (PIH) scale was designed to assess generic knowledge, attitudes, behaviors, and effects of self-management among patients with chronic diseases (57). The PIH scale is a 12-item tool consisting of 3 subscales, namely, knowledge, coping, and adherence/management subscales. Higher scores indicate better self-management. The internal consistency (Cronbach’s alphas ranged from 0.773 to 0.845) was good. The test-retest reliability was high (ICC = 0.818). The scale was used to evaluate criterion validity.

#### Mental Health Self-management Questionnaire

The original MHSQ examines how often people used self-management strategies in the last 2 months. The MHSQ consists of 18 items covering clinical (finding resources and getting help), empowerment (building upon their strengths and a positive self-concept), and vitality (adopting a healthy and active lifestyle) subscales. Each item is rated on 5-point Likert scales ranging from 0 (never used) to 4 (very often used). The reliability and validity of the MHSQ were adequate (45).

### Statistical analysis

The statistical analysis was performed with IBM SPSS software (version 26.0). Descriptive statistics (frequencies, percentages, means and standard deviations) were used for the sociodemographic and clinical variables.

For item analysis, the total MHSQ-C scores were arranged in descending and ascending order, with the first 27% being the high-level group and the last 27% being the low-level group. We used an independent samples *t*-test to compare item scores between the high-level and low-level groups. Entries with critical ratio values less than 3 could be considered for deletion (52). Pearson correlation analysis was used between the score of each item and the total MHSQ-C score. Items with an item-total correlation coefficient of less than 0.3 were considered for deletion from the scale (52, 58). We also performed a homogeneity test by Cronbach’s alpha, communalities and factor loading. An item was considered for deletion if it failed to meet the criteria more than 3 times in the above 5 statistical methods.

For the reliability assessment, a Cronbach’s alpha coefficient greater than 0.70 was considered reliable (59, 60). The test-retest reliability was evaluated by ICC, with a value of 0.7 or more considered acceptable (61).

For the validity evaluation, exploratory factor analysis (EFA) was performed to verify the construct validity. The Kaiser–Meyer–Olkin (KMO) test of sampling adequacy and Bartlett’s chi-square test of sphericity were conducted to confirm the suitability of the data for factor analysis. The KMO value was no less than 0.6, and the p value of Bartlett’s chi-square test of sphericity was less than 0.05, which was considered adequate to conduct EFA (46). EFA was performed by principal component analysis (PCA) with varimax rotation, and the factor loading was required to be more than 0.4. The content validity was assessed by a content validity index (CVI) using ratings of item relevance by experts (62). The interrater reliability/interrater agreement (IR) among experts was evaluated before computing the CVI, and the value of IR needed to be greater than 0.7. The item-level content validity index (I-CVI) was required to be no less than 0.78, which was the proportion of content experts that gave an item a relevance rating of 3 or 4 (63). The value of the average of the I-CVIs for all items on the scale (S-CVI/Ave) needed to be no less than 0.90 (63). The criterion validity was evaluated by Pearson relation analysis between the PIH scale and the MHSQ-C. Pearson’s correlation coefficients of 0.40–0.70 and >0.70 were considered moderate and strong, respectively, while 0.20–0.40 and <0.20 were considered weak and poor correlations, respectively (46). We proposed that the “gold standard” correlation was at least 0.70 (60).

### Procedure

This study was a part of a randomized controlled trial examining self-management in patients with mental illness. The whole study was approved by the biomedical ethics committee (number: 2019-0961), and the clinical trial registration number was ChiCTR1900028410. Prior to study initiation, oral consent was obtained from the subjects. Additionally, for minors, consent from their guardians was obtained. The participants were informed that they could refuse or stop participating without penalty. The data were processed confidentially.

## Results

### Development of the Chinese version of the Mental Health Self-management Questionnaire

Some expressions were found to be problematic during the translation process. Therefore, the expressions were reviewed and modified in the course of the forward translation and reconciliation, back translation and harmonization, and expert committee review steps. The resulting modifications were incorporated into an interim Chinese version of the MHSQ. In general, the terms “professionals” and “healthcare professionals” were combined, and other terms, such as, “sports” and “physical activity,” were suggested for clarification. Additionally, in the course of back translation and expert committee review, item 8 (“I learn to differentiate between my mental health problem and myself as a person”) was found to be difficult to understand. Therefore, discussion was necessary to determine how to translate “myself as a person.” Considering the suggestions of the expert committee and the results of pretesting among patients, item 8 was deleted from the Chinese version.

### Participant characteristics

A total of 440 participants were screened, 384 were recruited (56 participants were excluded according to the inclusion and exclusion criteria), and 326 participants (28 participants refused, 30 participants withdrew from the study) were used for analysis. The mean age of the participants was 34.23 (13–78, SD = 15.55). The ratio of outpatients to inpatients was approximately 1:1 (162 outpatients: 164 inpatients). The mean total MHSQ score was 38.41 (7–66, SD = 11.21). Details about the sociodemographic and clinical characteristics of the participants are shown in Table 1.

**TABLE 1:** Sociodemographic and clinical characteristics of the participants (*N* = 326).

Characteristics	n (mean)	% (SD)
Age (years)	34.23	15.55
**Gender**		
Male	101	31.0
Female	225	69.0
**Ethnicity**		
Han	307	94.2
Other	19	5.8
**Marital status**		
Unmarried	151	46.3
Married	154	47.2
Divorced or widowed	21	6.4
**Educational level**		
Primary school or below	12	3.7
Junior high school	44	13.5
High school	92	28.2
Junior college	73	22.4
Bachelor’s degree or above	105	32.2
**Per capita monthly household income (RMB)**		
≤1000	11	3.4
1001–3000	60	18.4
3001–5000	85	26.1
5001–7000	82	25.2
≥7001	88	27.0
**Medical insurance**		
No	130	39.9
Yes	196	60.1
**Place of residence**		
Rural	30	9.2
Urban or town	296	90.8
**Diagnosis**		
F31 Bipolar affective disorder	53	16.3
F32 Depressive episodes	96	29.4
F33 Recurrent depressive disorders	28	8.6
F40 Phobic anxiety disorders	2	0.6
F41 Other anxiety disorders	**147**	**45.1**
F41.0 Panic disorder	7	2.1
F41.1 Generalized anxiety disorder	26	8.0
F41.2 Mixed anxiety and depressive disorder	37	11.3
F41.9 Anxiety disorder, unspecified	77	23.6
**PHQ-9**		
No depression (0–4)	60	18.4
Mild depression (5–9)	73	22.4
Moderate depression (10–14)	54	16.6
Moderately severe depression (15–19)	68	20.9
Severe depression (≥20)	71	21.8
**GAD-7**		
Minimal anxiety (0–4)	94	28.8
Mild anxiety (5–9)	69	21.2
Moderate anxiety (10–14)	83	25.5
Severe anxiety (≥15)	80	24.5
**Number of recurrences**		
[0,1]	199	61.0
(1,3]	96	29.4
>3	31	9.5
**Number of hospitalizations**		
[0,1]	203	62.3
(1,3]	92	28.2
>3	31	9.5
**The period from diagnosis to investigation (months)**		
[3,12]	141	43.3
(12,36]	76	23.3
>36	109	33.4
**Somatic comorbidities**		
Yes	67	20.6
No	259	79.4
**Mental comorbidities**		
Yes	40	12.3
No	286	87.7

PHQ-9, Patient Health Questionnaire-9; GAD-7, General Anxiety Disorder-7.

### Item analysis

As shown in Table 2, the critical ratio values were higher than 3 (6.16 to 18.52, *p* < 0.05) for all 17 items. Each item-total correlation was significant (*p*-value of all items <0.001) with the total MHSQ score and a correlation coefficient greater than 0.3. The homogeneity test (Table 2) showed that only item 2 (“I consult with a professional (a physician, psychologist, social worker, etc.) for my mental health problem”) failed to meet the criteria 3 times. Item 2, concerning the strategy of patient management of clinical symptoms, had important clinical significance; thus although the homogeneity test was not perfect, after discussion with experts in the research group, we decided to keep this item.

**TABLE 2:** Item analysis results of the Chinese version of the Mental Health Self-management Questionnaire (MHSQ-C).

Item	CR	Item-total correlation(r)	Homogeneity test		
			Cronbach’s Alpha if item deleted	Communalities	Factor loading
1. I look for available resources to help me with my difficulties (websites, organizations, healthcare professionals, books, etc.)	8.701***	0.481***	0.870	0.185^†^	0.430^†^
2. I consult with a professional (a physician, psychologist, social worker, etc.) for my mental health problem.	6.157***	0.383***	0.875^†^	0.082^†^	0.287^†^
3. I get actively involved in my follow-up with the healthcare professionals I consult (physician, psychologist, social worker, etc.).	9.094***	0.476***	0.873	0.141^†^	0.376^†^
4. I participate in a support or help group in order to help me manage the difficulties I’m experiencing.	7.097***	0.443***	0.874	0.130^†^	0.361^†^
5. I take medication for my mental health problem, following the indications of a healthcare professional.	7.612***	0.450***	0.871	0.190 ^†^	0.436 ^†^
6. I try to solve my difficulties one step at a time.	15.159***	0.696***	0.862	0.506	0.711
7. I try to recognize the warning signs of a relapse of my mental health disorder.	7.746***	0.470***	0.872	0.178 ^†^	0.422 ^†^
9. I focus my attention on the present moment.	12.812***	0.607***	0.865	0.418	0.646
10. I learn to live with my strengths and weaknesses.	14.420***	0.704***	0.861	0.569	0.754
11. I congratulate myself on my successes, whether small or large.	17.647***	0.704***	0.860	0.547	0.740
12. I try to love myself as I am.	14.635***	0.696***	0.861	0.540	0.735
13. I take my capabilities into account when arranging my schedule.	8.650***	0.529***	0.868	0.293	0.542
14. I find comfort and an attentive ear in the people around me.	8.115***	0.499***	0.870	0.239	0.488
15. I engage in activities I like in order to maintain an active life.	18.520***	0.732***	0.859	0.583	0.764
16. I engage in sports, physical activity.	15.977***	0.662***	0.863	0.485	0.696
17. I have a healthy diet.	11.743***	0.614***	0.865	0.402	0.634
18. I do exercises to relax (yoga, tai-chi, breathing techniques, etc.).	13.822***	0.643***	0.864	0.444	0.666
Criteria	CR ≥ 3.00	*r* ≥ 0.300	0.874 ^‡^	≥0.200	≥0.450

MHSQ-C, Chinese version of the Mental Health Self-management Questionnaire; CR, Critical Ratio. ***p < 0.001.

^†^The values did not meet the criteria.

^‡^The Cronbach’s alpha of the MHSQ-C was 0.874.

### Reliability analysis

The Cronbach’s α coefficient for the whole scale of 17 items was 0.874, that for the clinical subscale was 0.706, that for the empowerment subscale was 0.818, and that for the vitality subscale was 0.830 (Table 3). A total of 37 patients were retested for test-retest reliability at a mean interval of 9.35 days (SD = 2.04, range = 7–14). The ICCs of MHSQ-C, clinical, empowerment and vitality subscales were 0.783 (95% confidence interval (CI) [0.616, 0.882]), 0.525 (95% CI [0.240, 0.725]), 0.786 (95% CI [0.622, 0.884]), and 0.748 (95% CI [0.564, 0.862]), respectively.

**TABLE 3:** Internal consistency and test-retest reliability results.

	Mean ± SD *n* = 326	Cronbach’s α coefficient *n* = 326	Test^†^ *n* = 37	Retest^†^ *n* = 37	ICC 95% CI *n* = 37
Total MHSQ-C	38.41 ± 11.21	0.874	34.95 ± 10.78	37.11 ± 12.27	0.783 [0.616, 0.882]
Clinical	10.93 ± 3.78	0.706	10.68 ± 3.86	12.30 ± 4.23	0.525 [0.240, 0.725]
Empowerment	18.91 ± 6.15	0.818	17.30 ± 5.95	17.51 ± 6.74	0.786 [0.622, 0.884]
Vitality	8.57 ± 3.82	0.830	6.97 ± 3.63	7.30 ± 3.44	0.748 [0.564, 0.862]

^†^The values are means and standard deviations.

MHSQ-C, Chinese version of the Mental Health Self-management Questionnaire; SD, standard deviation; ICC, intraclass correlation coefficient; CI, confidence interval.

### Validity analysis

#### Ceiling, floor effects, and skewness

**FIGURE 2:**
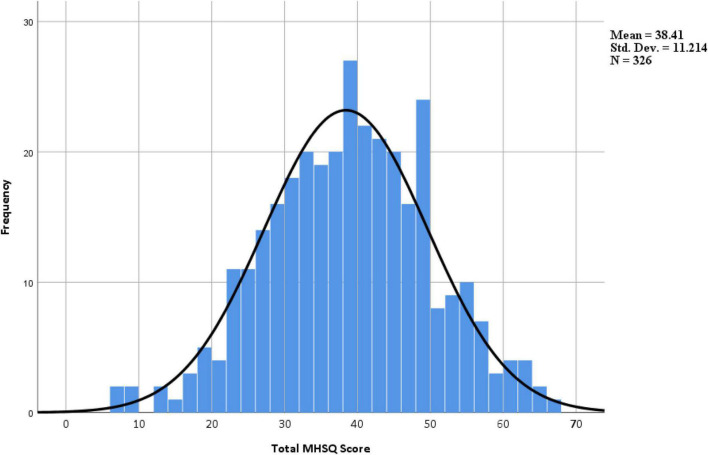
Histogram of the MHSQ score. MHSQ, Mental Health Self-management Questionnaire.

The skewness and kurtosis of the MHSQ were −0.085 and −0.110, respectively. The total scores of the MHSQ were well distributed with a range from 7 to 66 (Figure 2), which means there were no floor or ceiling effects.

#### Content validity

In this study, the content validity of the questionnaire was evaluated by expert committee review. The expert panel consisted of 9 experts in the fields of clinical nursing, psychological nursing, psychiatry and mental health. Specific information about the experts is shown in Supplementary Appendix B. The IR among experts was 0.89, I-CVI values were 0.89 to 1.00, and S-CVI/Ave was 0.99.

#### Factor analysis

The result for the KMO measure of sampling adequacy was 0.885, with significant Bartlett’s chi-square test of sphericity (*x*^2^ = 2,123.552, df = 136, *p* < 0.001). PCA and varimax rotation extracted three factors, which explained 53.68% of the total variance. The three-factor structure indicated by the Scree plot was optimal. Table 4 shows the whole factor loading. Factor 1 included 5 items measuring active and healthy lifestyles, factor 2 was composed of 7 items measuring how to build on strengths and positive self-concept to gain control, and factor 3 included 5 items measuring how to obtain help and use resources. Additionally, three items (items 6, 10, and 15) loaded onto factor 1 and factor 2 with high factor loadings (>0.40). Considering the original scale structure and factor loadings, items 6 and 10 were placed in factor 2, and item 15 was placed in factor 1.

**TABLE 4:** Factor analysis and mean scores of the MHSQ-C.

Item	Mean	SD	Factor loading		
			Factor 1	Factor 2	Factor 3
1. I look for available resources to help me with my difficulties (websites, organizations, healthcare professionals, books, etc.)	2.19	1.064	0.156	0.204	0.529
2. I consult with a professional (a physician, psychologist, social worker, etc.) for my mental health problem.	2.23	1.092	−0.082	0.089	0.778
3. I get actively involved in my follow-up with the healthcare professionals I consult (physician, psychologist, social worker, etc.).	1.94	1.304	0.042	0.077	0.813
4. I participate in a support or help group in order to help me manage the difficulties I’m experiencing.	1.23	1.251	0.190	0.005	0.626
5. I take medication for my mental health problem, following the indications of a healthcare professional.	3.34	0.879	−0.048	0.531	0.344
6. I try to solve my difficulties one step at a time.	2.79	0.994	0.446	0.449	0.335
7. I try to recognize the warning signs of a relapse of my mental health disorder.	2.55	1.159	0.257	0.110	0.486
9. I focus my attention on the present moment.	2.24	1.132	0.586	0.392	0.001
10. I learn to live with my strengths and weaknesses.	2.23	1.126	0.498	0.649	0.005
11. I congratulate myself on my successes, whether small or large.	2.01	1.301	0.343	0.747	0.078
12. I try to love myself as I am.	2.10	1.203	0.371	0.740	0.027
13. I take my capabilities into account when arranging my schedule.	2.71	1.016	0.140	0.643	0.093
14. I find comfort and an attentive ear in the people around me.	2.29	1.184	0.140	0.540	0.128
15. I engage in activities I like in order to maintain an active life.	2.06	1.162	0.677	0.402	0.137
16. I engage in sports, physical activity.	2.23	1.230	0.866	0.122	0.098
17. I have a healthy diet.	2.49	1.052	0.611	0.226	0.214
18. I do exercises to relax (yoga, tai-chi, breathing techniques, etc.).	1.79	1.251	0.779	0.137	0.148

MHSQ-C, Chinese version of the Mental Health Self-management Questionnaire; SD, standard deviation.

Extraction Method: Principal Component Analysis. Rotation Method: Varimax with Kaiser Normalization.

Factor 1: Vitality subscale; Factor 2: Empowerment subscale; Factor 3: Clinical subscale.

Compared with the original version of the MHSQ, item 5 shifted from the clinical subscale (factor loading = 0.34) in the original MHSQ to the empowerment subscale (factor loading = 0.53) in the current Chinese version, item 7 shifted from the empowerment subscale (factor loading = 0.43) to the clinical subscale (factor loading = 0.49), and item 9 shifted from the empowerment subscale (factor loading = 0.71) to the vitality subscale (factor loading = 0.59).

#### Criterion validity

The criterion validity showed that the correlation coefficient between the MHSQ-C and PIH scale was 0.738 (*p* < 0.001), and the correlation coefficient of subscales and PIH scale was 0.429 for the clinical subscale, 0.673 for the empowerment subscale, and 0.659 for the vitality subscale.

## Discussion

The study aimed to develop and verify the reliability and validity of the Chinese version of the MHSQ. The results of the psychometric properties of the MHSQ-C indicated satisfactory internal consistency, test-rest reliability, content validity, and criterion validity and adequate construct validity.

### The Chinese version of the Mental Health Self-management Questionnaire development process

We carried out the development procedures of the MHSQ-C in strict accordance with the guidelines and principles of Wild et al. (48) and Beaton et al. (49). The translation project was conducted by a team consisting of one native Chinese speaking coordinator proficient in English, two native Chinese speaking translators proficient in English, two native English speaking back translators proficient in Chinese and two clinical experts. During translation and language verification, the expressions were modified to produce an easy-to-understand questionnaire that included grammatically correct and natural Chinese and remained conceptually identical to the original English version.

In the cross-cultural adaptation stage, the MHSQ-C was modified by expert committee review and pretesting with the aim of ensuring consistency with Chinese cultural and language expression. Item 8 was deleted from the Chinese version after expert committee review and pretesting among patients. Item 8, with its emphasis on distinguishing “myself as a person” from “my mental health problem,” was difficult for patients to understand. More than 80% of patients did not understand the item in the pretesting stage. The expert committee held that this item was not appropriate for the Chinese cultural context. Most Chinese people were not good at expressing their feelings about themselves and had difficulty being aware of the “self as a person,” so it was recommended that the item be deleted. Translating questionnaires into other languages requires careful consideration of cultural and target population differences, which is not a simple process. Overall, the final Chinese version in this study was confirmed as conceptually equivalent to the original English version.

### Psychometric properties of the Chinese version of the Mental Health Self-management Questionnaire

In this study, the MHSQ-C showed good reliability in terms of Cronbach’s α coefficients and ICCs. First, the Cronbach’s α coefficients of the whole scale (0.874) and three subscales (0.706 for the clinical subscale, 0.818 for the empowerment subscale, and 0.830 for the vitality subscale) were high, which indicates good internal consistency (59, 60). Moreover, the ICCs of the total scale, empowerment subscale and vitality subscale (0.783, 0.786, 0.748, respectively) showed good test-retest reliability, and the ICC of the clinical subscale (0.525) was acceptable but relatively low. The clinical subscale includes five items referring to receiving help and using resources (45). This result may be related to participants’ characteristics. The test-retest sample population included both outpatients and inpatients, and inpatients might have changed their help-seeking behaviors as a result of receiving treatment, resulting in lower test-retest reliability of the clinical subscale.

Regarding the validity of the MHSQ-C, the IR among experts was 0.89 (I-CVI range = 0.89–1.00; S-CVI/Ave = 0.99), which indicated good content validity (63). The EFA results showed that the three-factor structure explained 53.68% of the total variance, which was higher than the Japanese version (47.83%) (46). The translated Chinese version with 17 items consists of three factors (clinical, empowerment and vitality), which is similar to the original questionnaire developed by Coulombe et al. (45). The subtle differences were that items 5 and7 exchanged their attributes with each other, and item 9 switched from the empowerment subscale to the vitality subscale. This may be due to cultural differences that cause patients to understand some items differently than the original questionnaire. In summary, the structure obtained from the EFA was generally consistent with that of the original questionnaire, indicating that the MHSQ-C has a reasonable structure. Concerning the criterion validity, the correlation coefficients between the MHSQ-C, and the empowerment subscale, vitality subscale and PIH scale (0.738, 0.673, and 0.659, respectively) indicated satisfactory criterion validity (60), and the correlation coefficient between the clinical subscale and the PIH scale (0.429) was acceptable (46). This may be due to the choice of a “gold standard.” In fact, there is no “gold standard” scale for assessing self-management skills among patients with mental illness, so we chose a self-management scale applicable to patients with chronic illness. The differences in how patients with mental illness and patients with chronic illness seek help and utilize resources led to the lower validity of the clinical subscale. A ceiling or floor effect concerns the proportion of respondents who achieve the highest or the lowest possible score. A floor or ceiling effect of 15% is considered the maximum acceptable (60). In this study, no floor or ceiling effect was observed. Overall, the MHSQ-C has good validity.

## Limitations

There were some limitations in this study. One limitation concerns the sample. Although we controlled for homogeneity, the sample population was from both outpatient and inpatient units, which may have had an impact on the study. This could be further validated in separate outpatient and inpatient populations. The other limitation concerns the questionnaire used to determine criterion validity. The PIH scale is not the “gold standard” for verifying criterion validity, although it is a relatively good option. Further studies can measure the psychometric properties among different psychiatric disorder populations or conduct different psychometric analyses, such as confirmatory factor analysis.

## Conclusion

This study provides preliminary evidence of the psychometric properties of the MHSQ-C in evaluating self-management strategies among people with mood and anxiety disorders. The MHSQ-C showed good reliability and validity, which will facilitate the development of self-management programs in China. The MHSQ-C may be used to conduct a comprehensive assessment of self-management behaviors and help individuals better understand what to do to strengthen their self-management skills.

## Data availability statement

The data supporting the findings of this study are available from the corresponding author upon reasonable request.

## Ethics statement

The studies involving human participants were reviewed and approved by the Biomedical Ethics Committee of West China Hospital, Sichuan University. Written informed consent from the participants’ legal guardian/next of kin was not required to participate in this study in accordance with the national legislation and the institutional requirements.

## Author contributions

MW, JW, XH, YW, and YH recruited the participants and performed the data collection. MW conducted the data analysis and drafted the manuscript. JW, XH, YH, JH, XL, and YF reviewed the manuscript. XL administrated and supervised the study. All authors contributed to the study’s conception and design and have agreed on the final version.
