# Supplementary material for: Reliability and validity of the Mental Health Self-management Questionnaire among Chinese patients with mood and anxiety disorders
Source: Front Psychiatry. 2022 Jul 29;13:952951. doi: 10.3389/fpsyt.2022.952951 (PMC9372341; doi:10.3389/fpsyt.2022.952951)
Supplement: Supplementary file 1 [file Data_Sheet_1.pdf]

Appendices

Appendix A. The English version of Mental Health self-management Questionnaire†

Items	Response				
	Never	Very rarely	Rarely	Often	Very often
**CLINICAL**					
1. I look for available resources to help me with my difficulties (websites, organizations, healthcare professionals, books, etc.)					
2.I consult with a professional (a physician, psychologist, social worker, etc.) for my mental health problem.					
3. I get actively involved in my follow-up with the healthcare professionals I consult (physician, psychologist, social worker, etc.).					
4. I participate in a support or help group in order to help me manage the difficulties I'm experiencing.					
5. I take medication for my mental health problem, following the indications of a healthcare professional.					
**EMPOWERMENT**					
6. I try to solve my difficulties one step at a time.					
7. I try to recognize the warning signs of a relapse of my mental health disorder.					
8. I learn to differentiate between my mental health problem and myself as a person.					
9. I focus my attention on the present moment.					
10. I learn to live with my strengths and weaknesses.					
11. I congratulate myself on my successes, whether small or large.					
12. I try to love myself as I am.					
13. I take my capabilities into account when arranging my schedule.					
14. I find comfort and an attentive ear in the people around me.					
**VITALITY**					
15. I engage in activities I like in order to maintain an active life.					
16. I engage in sports, physical activity.					
17. I have a healthy diet.					
18. I do exercises to relax (yoga, tai-chi, breathing techniques, etc.).					

†The English version of Mental Health self-management Questionnaire was obtained from the author of MHSQ by email, all use must be approved by the author.

Appendix B. The expert's information during the development of the Chinese version of Mental health self-management questionnaire.

Groups	Gender	Age	Nationality	Education level	Language background	Major	Years of professional experience
**Forward translation**	Male	47	Chinese	Medicine Doctor	Visiting Scholar Abroad	Cognitive and Clinical Psychology	25
	Female	50	Chinese	Master degree	Visiting Scholar Abroad	Medical education in English	27
**Back translation**	Female	55	Chinese American	Master degree	Visiting Scholar Abroad	Licensed Nursing Home Administrator	20
	Male	33	Chinese	Doctor degree	Scholar Abroad	Intimate Partner Violence	10+
**Expert committee**	Male	47	Chinese	Medicine Doctor	/	Cognitive and Clinical Psychology	25
	Male	33	Chinese	Doctor degree	/	Intimate Partner Violence	10+
	Female	37	Chinese	Doctor degree	/	Psychiatric Nursing	10+
	Female	47	Chinese	Bachelor's degree	/	Psychiatric Nursing and Psychological Counseling	27
	Male	40	Chinese	Doctor degree	/	Psychological Counseling and Nursing Management	8
	Male	46	Chinese	Doctor degree	/	Psychosomatic Medicine and Scale Research	22
	Male	45	Chinese	Doctor degree	/	Psychosomatic Medicine	21
	Female	40	Chinese	Bachelor's degree	/	Psychological Counseling	17
	Female	33	Chinese	Doctor degree	/	Psychiatric Nursing	10
